# An In Vitro Estimation of the Cytotoxicity and Genotoxicity of Root Extract from *Leonurus sibiricus* L. Overexpressing AtPAP1 against Different Cancer Cell Lines

**DOI:** 10.3390/molecules23082049

**Published:** 2018-08-16

**Authors:** Przemysław Sitarek, Ewelina Synowiec, Tomasz Kowalczyk, Tomasz Śliwiński, Ewa Skała

**Affiliations:** 1 Department of Biology and Pharmaceutical Botany, Medical University of Łódź, Muszyńskiego 1, 90-151 Łódź, Poland; ewa.skala@umed.lodz.pl; 2 Laboratory of Medical Genetics, Faculty of Biology and Environmental Protection, University of Łódź, Pomorska 141/143, 90-236 Łódź, Poland; ewelina.synowiec@biol.uni.lodz.pl (E.S.); tomasz.sliwinski@biol.uni.lodz.pl (T.Ś.); 3 Department of Genetics and Plant Molecular Biology and Biotechnology, The University of Łódź, Banacha 12/13, 90-237 Łódź, Poland; tomasz.kowalczyk@biol.uni.lodz.pl

**Keywords:** AtPAP1 transgenic roots of *Leonurus sibiricus*, cytotoxic effect, mtDNA copy number, mitochondrial membrane potential, genotoxic effect

## Abstract

As the current cancer treatment success rate is not sufficient, interest has grown in plants as possible sources of anti-cancer compounds. One such plant with a broad spectrum of activity is *Lenourus sibiricus* of the family Lamiaceae. This study investigates for the first time both the genotoxic and cytotoxic activities of TR (transformed) and AtPAP1 TR (with over-expression of transcriptional factor) root extracts of *Lenourus sibiricus* against various cancer cell lines (CCRF-CEM, K-562 and A549). Both tested extracts showed a cytotoxic effect on CCRF-CEM and K-562 cell lines, but strongest activity was observed for the AtPAP1 TR extract. No cytotoxic effect was observed against the A549 cell line in the tested concentration range, and it was found that both tested extracts may induce apoptosis by decreasing mitochondrial membrane potential and inducing nDNA damage lesion in the *TP53* region and mtDNA in *ND1* (mitochondrially encoded NADH: ubiquinone oxidoreductase core subunit 1) and *ND5* (mitochondrially encoded NADH:ubiquinone oxidoreductase core subunit 5) regions in K-562 and CCRF-CEM. Our results confirmed that TR and AtPAP1 TR root extracts from *L. sibiricus* are cytotoxic and genotoxic against different model cell lines (CCRF-CEM and K-562)*.* However, the observed genotoxicity of both extracts needs to be confirmed by additional studies. These preclinical observations support the use of *L. sibiricus* with other pharmacological purposes.

## 1. Introduction

Cancer remains one of the leading causes of morbidity and mortality globally, following cardiovascular disease [1]. As chemotherapy regimens can put patients under considerable strain and further damage their health, there is a focus on using alternative treatments [2,3]. Medicinal plants, and the natural biologically-active compounds they contain, have played a significant role in drug discovery and offer potential for development as therapeutic agents [4]; however, of approximately more than 300,000 plant species reported, only a small percentage has been the subject of phytochemical and biological activity studies [5]. A great part of the pharmaceuticals available are still derived from natural sources, and there is growing world-wide interest in the use of phyto-pharmaceuticals in modern medicine [6,7]. In the past few years, considerable advancements have been made in the natural products endowed with antimutagenic and anticarcinogenic properties. Additionally, plant derived anti-cancer agents are known to be safer and give fewer side effects than available synthetic anti-cancer agents [8]. Natural compounds known as dietary chemopreventive products offer a great potential in the fight against cancer through various mechanisms, including antioxidant activity, antimutagenic activity, enzyme modulation, gene expression and apoptosis, which have been tested in various cellular models [9]. 

*Leonurus sibiricus* L. is an aromatic medicinal plant in the Lamiaceae family used by folk practitioners in Bangladesh, China, India and elsewhere against a range of diseases including asthma, cardiovascular, diabetes or menstrual irregularities. Material obtained from *L. sibiricus* possesses varied pharmacological properties such as antioxidative, anti-inflammatory, antimicrobial, hepatoprotective and anticancer effects [10,11,12,13,14]. As *L. sibiricus* presents great variability in terms of biological activities, it represents an interesting source of active secondary metabolites to be used for their potential antitumor activities. *L. sibiricus* root extract has been reported to have cytotoxic activity against glioma cells and melanoma cells, which was attributed to the presence of higher levels of phenolic compounds including caffeic acid, chlorogenic acid, ferrulic acids and *p*-coumaric acid [15]. Additionally, further genetic manipulations, such as transformation by *Agrobacterium* or the introduction of specific transcriptional factors, have resulted in elevated production of secondary metabolites such as phenolic acids and hence, further improved biological properties [11,13]. The regulation of apoptosis is of paramount importance in cancer biology, because large numbers of cancer cells are defective in this regard [16]. *L. sibiricus* extract has been found to induce apoptosis through intrinsic and extrinsic pathways [11], and by *via* DNA damage [17]. DNA damage will cause cell cycle arrest, possibly resulting in DNA repair or cell death *via* apoptosis [18].

Therefore, the present study investigates the cytotoxic and genotoxic activity of extract from *L. sibiricus* root with over-expression of transcriptional factor (AtPAP1 TR) and transformed roots (TR) against A549, CCRF-CEM and K-562 human cancer cell lines with regard to its effect on apoptosis.

## 2. Results

### 2.1. Determination of Phenolic Acids Content in AtPAP1 and TR Root Extracts by HPLC Analysis

All procedures describing the phenolic acid content in TR and AtPAP1 TR root extracts have been performed previously [15,17]. All phenolic acids (neochlorogenic acid, chlorogenic acid, caffeic acid, *p*-coumaric acid and ferulic acid) were present in greater amounts in the AtPAP1 TR root extract from the bioreactor than the TR roots. The major compound in both extracts was chlorogenic acid. The chlorogenic acid content in the AtPAP1 TR root extract from the bioreactor was 21,520 µg of dry weight, i.e., 5.24 times higher than in the TR root extract (4104 µg/g of dry weight) (*p* < 0.05). The second phenolic acid, i.e., caffeic acid, constituted 14,500 µg/g of dry weight in the AtPAP1 TR root extract from bioreactor, i.e., 3.47-times higher than that in the TR root extract (4176 µg/g of dry weight). Results are presented in Table 1.

### 2.2. Assessment of Cytotoxic Activity

**Figure 1:**
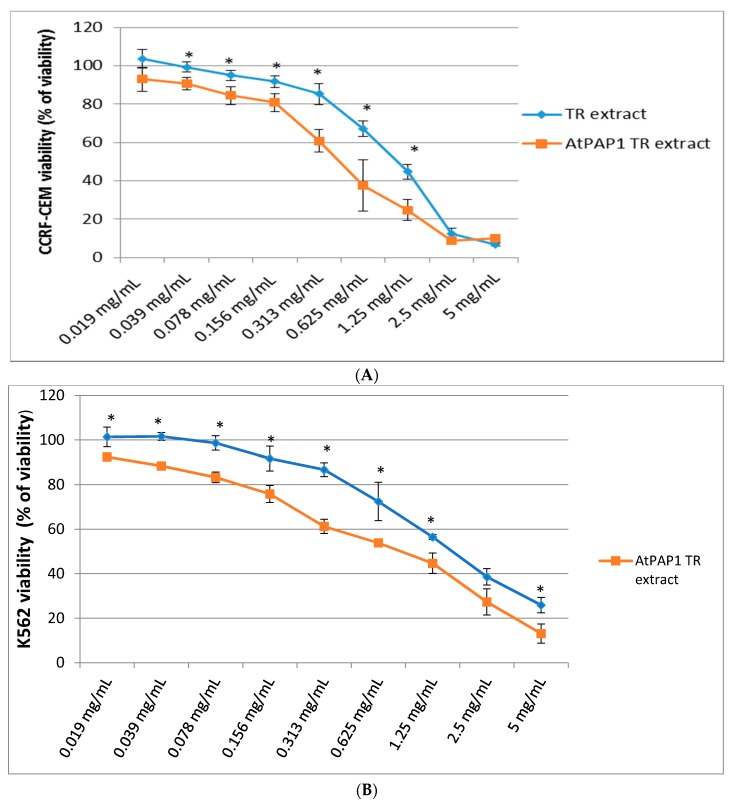

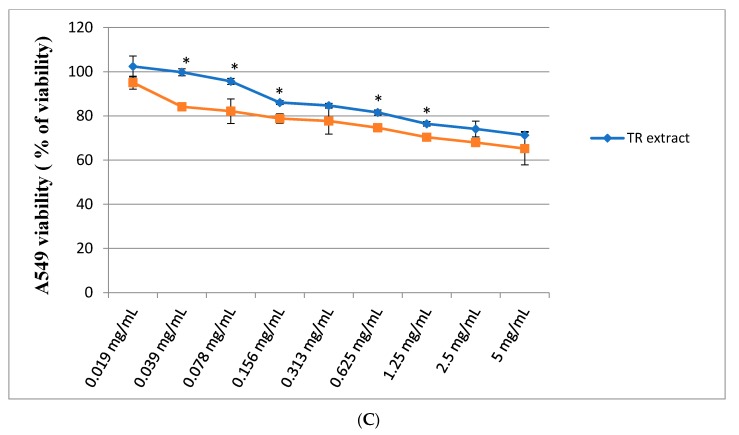
The effect of TR and AtPAP1 TR root extract of *L. sibiricus* (in concentrations of 0.019–5 mg/mL) on (**A**) CCRF-CEM, (**B**) K562 and (**C**) A549 cells viability. The data represent the mean of 3 measurements. *: *p* < 0.05 TR vs. AtPAP1 TR.

In this experiment we investigated the cytotoxic activity of the TR and AtPAP1 TR root extracts of *L. sibiricus* (in a concentration of 0.019–5 mg/mL) in three cell lines (K-562, A549 and CCRF-CEM), using MTT assay. The shape of the curve shows inhibition of cell proliferation in K-562 and CCRF-CEM cell lines (Figure 1A,B) after 24 h of treatment. In both cases, the TR root extract had a weaker cytotoxic effect than AtPAP1 TR root extract. For the CCRF-CEM cells, the LC_50_ was around 0.313 mg/mL of the AtPAP1 TR extract but around 1.25 mg/mL for the TR extract. In turn, for the K-562 cells, the LC_50_ was 0.625 mg/mL for of AtPAP1 TR but 1.25 mg/mL for TR. Additionally, neither the AtPAP1 TR nor the TR extracts demonstrated any cytotoxic effect (i.e., killing 50% cells) against A549 cells in the same concentration range (0.019–5 mg/mL). Results are presented in Figure 1C.

### 2.3. Effect of TR and AtPAP1 TR Root Extracts from L. sibiricus on Mitochondrial Membrane Potential Loss (ΛΨm) in K-562, CCRF-CEM and A549 Cells

**Figure 2:**
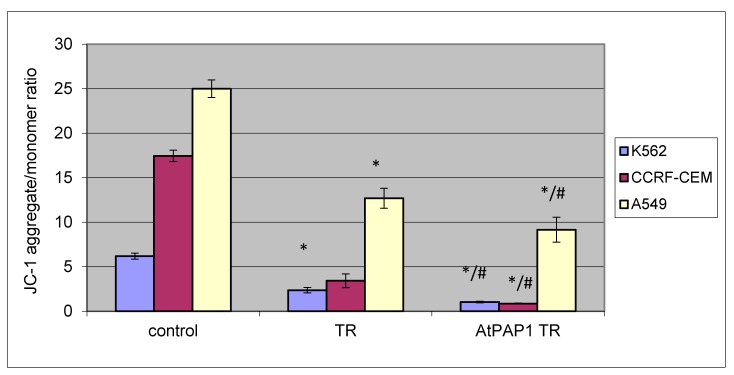
The effect of TR and AtPAP1 TR root extracts of *L. sibiricus* on mitochondrial membrane potential in CCRF-CEM, K562 and A549 cells. MMP is expressed as a ratio of 530 nm/590 nm to 485 nm/538 nm (aggregates to monomer) fluorescence, as quantified with a fluorescent plate reader after JC-1 staining. The data represent means ± standard deviation (SD). *: *p* < 0.05 Control vs. TR and AtPAP1 TR. ^#^: *p* < 0.05 TR vs. AtPAP1 TR.

The TR and AtPAP1 TR root *L. sibiricus* extracts induced a potent loss in ΛΨm after 24 h treatment in all tested cell lines (K-562, CCRF-CEM and A549) at concentration IC_50_. Compared to controls, the treated cells displayed decreased mitochondrial membrane potential. The AtPAP1 TR extract from bioreactor was found to be most potent. The effect of the TR and AtPAP1 TR extracts on ΛΨm is shown in Figure 2.

### 2.4. Mitochondrial Copy Number

**Figure 3:**
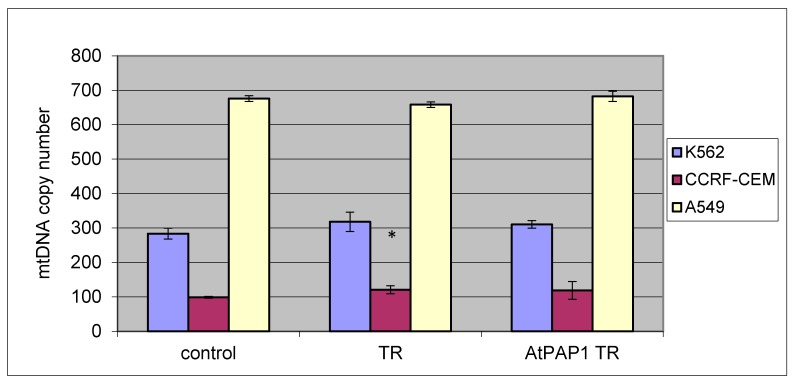
The effect of TR and AtPAP1 TR root extracts of *L. sibiricus* on mitochondrial DNA copy number in an CCRF-CEM, K562 and A549 cell line measured by real-time quantitative PCR. The data represent means ± SD. *: *p* < 0.05 Control vs. TR and AtPAP1 TR. ^#^: *p* < 0.05 TR vs. AtPAP1 TR.

Furthermore, no difference was found between treated and untreated cells, or between treated TR and AtPAP1 TR extracts, with regard to changes in mtDNA copy number for all tested cell lines (K-562, CRF-CEM and A549). Only a slight increase was observed in K-562 and CCRF-CEM without significant differences. Results are presented in Figure 3.

### 2.5. Quantification of Mitochondrial DNA (mtDNA) Damage

SLR-qRT-PCR amplification identified mtDNA damage of DNA isolated from A549, K-562 and CCRF-CEM cells exposed to TR and AtPAP1 TR root extracts for 24 h [19]. In the case of the CCRF-CEM and K-562 cell lines, a significant increase in lesion rate in the *ND1* region was found compared to controls. For the CCRF-CEM cells, about 7.8 lesions per 10 kb DNA in those treated with TR extract, nine lesions per 10 kb DNA after the AtPAP1 TR extract treatment. In turn for K-562 cells, about four lesions per 10 kb DNA in these cells treated with TR extract, six lesions per 10 kb DNA after AtPAP1 TR extract treatment. 

**Figure 4:**
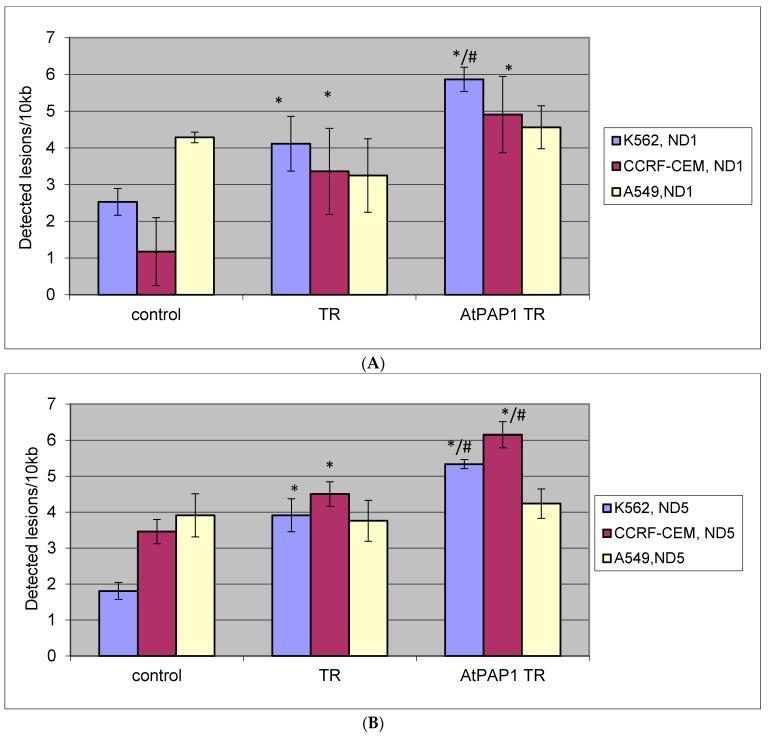
The effect of TR and AtPAP1 TR root extracts of *L. sibiricus* on mitochondrial DNA (mtDNA) lesion frequency per 10 kb DNA in (**A**) *ND1* and (**B**) *ND5* genes, estimated by SLR-qRT-PCR amplification of total DNA from CCRF-CEM, K562 and A549. The data represent means ± SD. *: *p* < 0.05 Control vs. TR and AtPAP1 TR. ^#^: *p* < 0.05 TR vs. AtPAP1 TR.

Regarding the *ND5* region, for CCRF-CE, about 4.5 lesions per 10 kb DNA were found in these cells treated with TR extract and 6.2 lesions per 10 kb DNA treated with AtPAP1 TR extract. For K-562, about 4.0 lesions per 10 kb DNA were found for the TR extract, 5.3 lesions per 10 kb DNA for the AtPAP1 TR extract. No significant differences were observed between tested extracts. For the A549 cells, no differences were found between TR and AtPAP1 TR extracts and controls with regard to the level of mtDNA damage in the *ND1* or *ND5* region. The results are shown in Figure 4A,B.

### 2.6. Quantification of Nuclear DNA (nDNA) Damage

The lesion rate was higher in the *TP53* region for K-562, CCRF-CEM cells after treatment with TR and AtPAP1 TR extracts. For K-562 cells, 3.1 lesions per 10 kb DNA was observed after treatment with TR extract, and 4.2 lesions per 10 kb DNA after AtPAP1 TR. For CCRF-CEM cells, 5.6 lesions per 10 kb DNA were found after treatment with TR extract and 6.9 lesions per 10 kb DNA after treatment with AtPAP1 TR extract.

**Figure 5:**
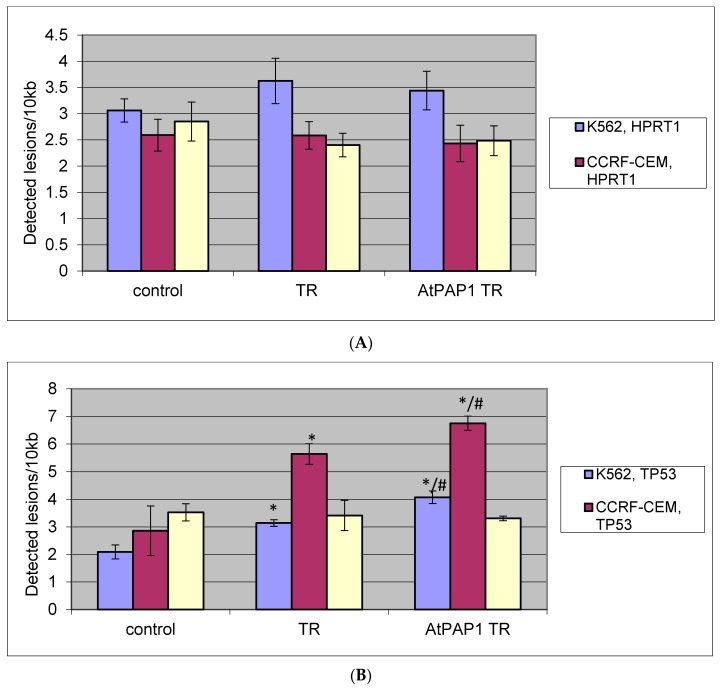
The effect of TR and AtPAP1 TR root extracts of *L. sibiricus* on nucelar DNA (nDNA) lesion frequency per 10 kb DNA in (**A**) *HPRT1* and (**B**) *TP53* genes, estimated by SLR-qRT-PCR amplification of total DNA from CCRF-CEM, K562 and A549. The data represent means ± SD. *: *p* < 0.05 Control vs. TR and AtPAP1 TR. ^#^: *p* < 0.05 TR vs. AtPAP1 TR.

For A549 cells, no differences were found between TR and AtPAP1 TR extracts and control regarding the level of nDNA damage in the *TP53* region. In the *HPRT1* region: no significant differences between TR and AtPAP1 TR extracts and controls were found regarding the amount of nDNA damage for K-562, CCRF-CEM or A549 cells. All results as shown in Figure 5A,B.

## 3. Discussion

Cancer-related research is conducted worldwide, since cancer is a leading cause of death. Studies on cancer often examine the effects of biologically-active substances on cancer cells, many of which originate from various plants [20,21] and there is a great need to better understand these reliable and inexhaustible sources. Plant biotechnology plays a large role in these studies as it can allow the cheap acquisition of active plant compounds. 

It is important to understand the mechanisms of anticancer agents for future application in cancer therapy [22,23,24]. Many important events during apoptosis are closely related to mitochondria, including the loss of mitochondrial membrane potential, the release of cytochrome C and apoptosis-inducing factor, and changes in the activity of Bcl-2 family proteins [25,26,27]. Our earlier studies showed that apoptosis can be induced by intrinsic and extrinsic pathways, and the loss of mitochondrial potential can be induced in various grades of glioma cells following treatment with *L. sibiricus* extracts [11]. Additionally, genetic manipulation (by *Agrobacterium rhizogenes* or over-expression AtPAP1 transcriptional factor) on this species can enhance the action of the extract by increasing the secondary metabolite production [13], and AtPAP1 TR roots grown in a 5 L bioreactor were found to be characterized by an even higher content of phenolic acids [15]. This method may be used in the future to obtain active compounds in the treatment of many diseases. 

The present study examines the cytotoxic and genotoxic effects of TR and AtPAP1 TR root extracts from *L. sibiricus* in different model cell lines (K-562, CCRF-CEM and A549). Cytotoxic effects were observed across a range of concentrations (0–5 mg/mL) in K-562 and CCRF-CEM cell lines after treatment of TR and AtPAP1 TR root extracts but the most effective was AtPAP1 TR root extract from bioreactor. These differences may result from the different content of active phenolic compounds such as chlorogenic acid, caffeic acid, ferrulic acid and *p*-coumaric acid. Literature reports that various polyphenols affect a range of cell functions including growth, differentiation and apoptosis [28], and they may inhibit cancer cells by xenobiotic metabolizing enzymes that alter metabolic activation of potential carcinogens [29]. The mechanism of action behind the anticancer activity of phenolics could act by disturbing cell division during mitosis at the telophase. It has also been reported that phenolics reduce the cellular protein level and mitotic index, as well as colony formation during cancer cell proliferation. In addition, the presence of a 2,3-double bond in flavonoid molecules correlates with mitochondrial damage and cancer cell death [30]. 

Our previous study found that *L. sibiricus* root extracts rich in phenolic compounds showed cytotoxic effects on glioma cells and melanoma cells [11,15] but not to have any cytotoxic effect on normal human astrocytes (NHA) [11]. Our present results indicate that the extracts do in fact demonstrate toxicity toward other cell lines (K-562 and CCRF-CEM) but not against A549 cell line. It is most likely that the negative result obtained might be due to heterogeneity in the sensitivity of different cancer cell lines to the phenolic content in *L. sibiricus* extracts. Our results are consistent with those of Czerwonka et al., which indicate that *Hordeum vulgare* water extract (HWE), rich in phenolic compounds, had a dose-dependent cytotoxicity against HT-29 cells, but not A549 cells [31]. 

The present report is the first to make a thorough examination of the cytotoxic activity of AtPAP1 and TR *L. sibiricus root extracts* on CCRF-CEM, K-562 and A549 cell lines. Mitochondria are the main sites of intracellular oxidative phosphorylation and adenosine triphosphate (ATP) synthesis. Changes in membrane phosphatidylserine play an important role in collapse of the mitochondrial membrane potential [32]. Our results indicate that the treatment of K-562, CCRF-CEM and A549 cells with TR and AtPAP1 TR root extracts decreased mitochondrial membrane potential, but better results were observed for the AtPAP1 root extract. Yen et al. revealed that *Ficus beecheyana* root extracts rich in phenolic acids (*p*-hydroxybenzoic acid, caffeic acid, chlorogenic acid, *p*-coumaric acid) may induce apoptosis by decreasing mitochondrial membrane potential in the HL-60 cell line [33]; however, Qi et al. report that *Athyrium multidentatum* extract, in which the major compound was chlorogenic acid, induces apoptosis via mitochondrial dysfunction in HepG2 cells [34]. Extracts from *L. sibiricus*, in which the most dominant compounds were the phenolic acids with chlorogenic acid and caffeic acid, have been found to decrease the mitochondrial membrane potential in glioma cells [11]. 

The present study also investigated the effect of TR and AtPAP1 TR root extracts from *L. sibiricus* on mitochondrial DNA by estimating the mitochondrial DNA copy number in the studies K-562, A549 and CCRF-CEM cell lines. They are also involved in multiple cellular processes such as cell differentiation, cell communication and cell apoptosis. The synthesis and degradation of mtDNA is rapid and independent of the cell cycle [35,36]. Due to the lack of protection from introns and histones, and less efficient DNA repair mechanisms, mtDNA is particularly susceptible to reactive oxidative species (ROS) and other sources of genotoxic damage, which may finally lead to sequence mutations or copy number alterations [37]. Changes in mtDNA copy number could alter the expression of mitochondrial genes and result in abnormal mitochondrial functions, such as energy production, signal transduction, apoptosis and cell growth [38]. Different signal transduction pathways interact with the mitochondria to influence apoptosis, but the role of mtDNA copy number in apoptosis has remained largely unexplored. Our present findings do not indicate any changes in mtDNA copy number after treatment of TR and AtPAP1 TR root extracts in any tested cell line. However, more mtDNA damage lesions were found in the *ND1* and *ND5* gene regions and more nDNA damage lesions in the *TP53* gene region. This is the first report to evaluate mtDNA copy number, mtDNA damage lesion number in the *ND1* and *ND5* genes region and nDNA damage lesions in *HPRT1* and *TP53* gene region after treatment of TR and AtPAP1 TR root extracts from *L. sibiricus*.

In contrast to our study, Bijak et al. revealed that polyphenolic compounds (silybin and silychristin) have a protective effect on cellular mitochondria, observed as a reduction of spontaneous mitochondrial DNA (mtDNA) damage in A549, measured as mtDNA copies, and mtDNA lesions in the *ND1* and *ND5* regions [39]. However, Mizumachi et al., found that the mtDNA copy number increased in drug-resistant tumor cells of the head and neck, and the increase in mtDNA copy number reduced the production of intracellular ROS [40]. Our previous study showed that TR and AtPAP1 extracts induce apoptosis *via* DNA damage and downregulation of selected epigenetic factors in glioma cells [17]. Basri et al. revealed that low doses of acetone extract from stem bark of *C. odontophyllum* containing polyphenols showed significant DNA damage in HCT 116 cells [41]. Similarly Arı et al. note that phenolic extract from *Pelargonium quercetorum* exerted genotoxic activity at relatively low doses in human breast cancer cells (MCF-7 and MDA-MB-231) [42]. 

The present study moves into new areas by examining the cytotoxic or genotoxic effects of *L. sibiricus* TR and AtPAP1 TR root extract on CCRF-CEM, K-562 and A549 cells. Therefore, further studies are needed to better understand the mechanisms behind these effects and the types of DNA lesion formed leading to apoptosis following exposure.

## 4. Materials and Methods

### 4.1. Establishment and Confirmation of L. sibiricus Transgenic Root Culture with Transcriptional Factor AtPAP1

The establishment and confirmation of the transformed root (TR) and AtPAP1 TR transgenic root (with transcriptional factor) were described in our earlier studies [11,13]. The AtPAP1 transgenic roots were grown in 5L bioreactor [15]. 

### 4.2. Plant Material and Extract Preparation

Lyophilized and powdered AtPAP1 TR roots and TR roots were used for analysis (10 g d.w.). The analysis was performed as described by Sitarek et al. [13]. The TR and AtPAP1 TR extracts were dissolved in PBS.

### 4.3. HPLC Analysis for Determination of Phenolic Acids

LC-MS/MS was used to identify the phenolic acids. The content of the phenolic acids in the AtPAP1 root extract was determined by HPLC according to Sitarek et al. [11]. 

### 4.4. Cell Cultures

All our experiments were performed on human lung adenocarcinoma A549 (CCL-185; ATCC) cell line, and the human T lymphoblast CCRF-CEM (CCL-119; ATCC) and chronic myelogenous leukemia K-562 (CCL-243; ATCC) cell lines. These cells were obtained from American Type Culture Collection (ATCC™, Manassas, VA, USA). The cell lines were maintained in an incubator with 5% CO_2_ atmosphere at 100% humidity and 37 °C in DMEM medium (A549) or RPMI 1640 medium (CCRF-CEM and K-562) supplemented with 10% (*v*/*v*) heat-inactivated Fetal Bovine Serum (FBS) and 100 U/mL penicillin, 100 μg/mL streptomycin. All cell culture media and components were purchased at Lonza (Basel, Switzerland).

### 4.5. Cell Viability

An MTT assay was employed to measure the viability of A549, CCRF-CEM and K-562 cells treated with different concentrations of TR and AtPAP1 TR root extracts from *L. sibiricus*. Briefly, cells were seeded at 1 × 10^4^ cells per well (A549) or 1 × 10^5^ cells (CCRF-CEM and K-562) per well in 96-well culture plates and allowed to grow overnight. The next day, the cells were incubated for 24 h with the tested extracts over a range of concentrations: 0 (control), 0.019, 0.039, 0.078, 0.156, 0.313, 0.625, 1.25, 2.5 and 5 mg/mL. After completing the incubation, the cells were washed once and incubated with 0.5 mg/mL of 3-(4,5-dimethylthiazol-2-yl)-2,5-diphenyl tetrazolium bromide (MTT) at 37 °C for 4 h. Thereafter, MTT was carefully removed and DMSO (100 μL) was added to each well and vortexed at low speed for five min to fully dissolve the blue crystals. Absorbance was measured at 570 nm with a reference at 630 nm using a Bio-Tek Synergy HT Microplate Reader (Bio-Tek Instruments, Winooski, VT, USA). Independent experiments were repeated in triplicate. Cell viability was expressed as a percentage relative to the untreated (control) cells, which was defined as 100%. 

### 4.6. Mitochondrial Membrane Potential (MMP)

MMP was determined by the fluorescent probe JC-1 (5′,6,6′-tetrachloro-1,1′,3,3′-tetraethylbenzimidazolylcarbocyanine iodide). Cells were seeded into black 96-well tissue culture plates with transparent bottom (Greiner Bio-One, Monroe, NC, USA) at a density of 1 × 10^4^ cells/well (A549), 1 × 10^5^ cells/well (CCRF-CEM and K-562) in 50 µL culture medium and allowed to grow overnight. The next day, cells were treated with indicated concentration IC_50_ of TR and AtPAP1 TR roots extracts for 24 h. Finally, the cells were preincubated with 5 μM JC-1 in the HBSS in a CO_2_ incubator at 37 °C for 30 min. Prior to measurements, the cells were centrifuged (300× *g* for 10 min at 22 °C) then washed twice with the HBSS. The fluorescence was measured on a Bio-Tek Synergy HT Microplate Reader with the filter pairs of 530 nm/590 nm and 485 nm/538 nm. Results are shown as a ratio of fluorescence, measured at 530 nm/590 nm to that measured at 485 nm/538 nm (aggregates to monomer fluorescence).

### 4.7. DNA Extraction from Cell Culture

Treated TR and AtPAP1 TR root extract cell suspensions (2 × 10^6^ cells) were collected by centrifugation and total genomic DNA (nuclear and mitochondrial) was isolated using the QIAamp DNA Mini Kit (Qiagen, Valencia, CA, USA) according to the manufacturer′s instructions. DNA concentrations were determined by spectrophotometric measurement of absorbance at 260 nm and the purities were calculated by A260/A280 ratio using Bio-Tek Synergy HT Microplate Reader. 

### 4.8. mtDNA Copy Number Quantification

Quantitative Real-Time PCR (qRT-PCR) was performed/used to determine relative amounts of mitochondrial DNA (mtDNA) and nuclear DNA (nDNA). Two primer pairs were used for detecting mtDNA (*ND1*, *ND5*) and two primer pairs for detecting nDNA (*SLCO2B1*, *SERPINA1*). Mitochondrial *ND1* (124 bp fragment size) and *ND5* (124 bp fragment size) genes were amplified using the primer pair (forward primer: 5′-CCTAAAACCCGCCACATCTA-3′ and reverse primer: 5′-GCCTAGGTTGAGGTTGACCA-3′; forward primer: 5′-AGGCGCTATCACCACTCTGT-3′ and reverse primer: 5′-TTGGTTGATGCCGATTGTAA-3′), respectively. For determination of the amount of nuclear DNA, the *SLCO2B1* (135 bp fragment size) and *SERPINA1* (148 bp fragment size) genes were used as a reference: forward primer: 5′-TGCAGCTTCCTCTTCACAGA-3′ and reverse primer: 5′-CTCAGCCCCAAGTATCTCCA-3′; forward primer: 5′-GATCCCAGCCAGTGGACTTA-3′ and reverse primer: 5′-CCTGAAGCTGAGGAGACAGG-3′. qRT-PCR amplification was performed using the CFX-96 detection system (Bio-Rad, Hercules, CA, USA) in 10 µL containing 1 × RT PCR Mix SYBR A (A&A Biotechnology, Gdynia, Poland), 250 nM each primer and 1 μL (5 ng) of genomic DNA. The thermocycling RT-PCR conditions used were as follows: 95 °С for 3 min, followed by 40 cycles at 95 °С for 15 s, 65 °С for 30 and 72 °С for 15 s with plate reading at this step. All primers were designed using Primer3 software (http://bioinfo.ut.ee/primer3-0.4.0/) and synthesized by Sigma-Aldrich (Steinheim, Germany). Complete nucleotide sequences for each gene taken from the ENSEMBL database. Each reaction was performed in duplicate and included a negative control (without template DNA). Relative quantification of the copy number of mtDNA using nDNA as a standard was determined using the following equation: 2^ΔCt1 and Ct2^, where ΔCt1 is the difference in the Ct values for the *ND1*/*SLCO2B1* pair (ΔCt1 = Ct for *SLCO2B1* − Ct for ND1); ΔCt2 is the difference in the Ct values for the *ND5*/*SERPINA1* pair (ΔCt2 = Ct for *SERPINA1* − Ct for *ND5*). 

### 4.9. Determination of Mitochondrial and Nuclear DNA Damage

The semi-long run quantitative RT-PCR (SLR-qRT-PCR) was used to assess mitochondrial DNA (mtDNA) and nuclear DNA (nDNA) damage, as described previously, with some modifications (Rothfuss [19]). SLR-qRT-PCR reactions were conducted in the CFX96^TM^ real-time system (Bio-Rad) and monitored by real-time measurement of the intercalation of the saturating fluorescent dye—SYBR Green to double-stranded DNA supplied by the RT PCR Mix SYBR A Kit (A&A Biotechnology). Cycling condition were as follows: initial denaturation of 3 min at 95 °C was followed by up to 40 cycles of 15 s at 95 °C, 30 s at 65 °C and 15 s at 72 °C (short fragments) or 45 s at 72 °C (long fragments). The SLR-qRT-PCR reaction mix consisted of 1 × RT PCR Mix SYBR A (A&A Biotechnology), 250 nM each primer and 1 ng of template DNA in a total volume of 10 µL per sample. To measure the levels of DNA lesion in tested region of the mitochondrial or nuclear genome, two fragments of different lengths, i.e., long and small fragments (served as internal undamaged reference), located in the same mitochondrial/nuclear genomic region were used. Details on the primers used for amplification of mitochondrial and nuclear loci are presented in Table 2. Data analysis was based on the measurement of Ct values derived from the SLR RT-PCR runs of the short and long amplicons. Ct values for the primer pair was obtained using the CFX Manager TM Software (version 3.1). DNA damage was calculated as lesion per 10kb DNA of each region by including the size of the respective long fragment:

$$\begin{array}{l} \mathrm{Lesion}\;\mathrm{per}\;10\;\mathrm{kb}\;\mathrm{DNA}\;\\ =\;\left(1\;-\;2{\;}^{-\left(\Delta long\;-\;\Delta short\right)}\right)\times \;10,000\;\left[bp\right]/size\;of\;long\;fragment\;\left[bp\right] \end{array}$$

where ∆*long* and ∆*short* indicate differences in Ct value between non-treated control and treated samples. DNA isolated from the non-treated cell (controls) was used as reference whereas Ct of the large and small mitochondrial/nuclear fragments were used for DNA damage quantification.

## 5. Statistical Analysis

All experiments were performed in triplicate. The results are expressed as mean ± SD. The Shapiro-Wilk test was used to verify the normality of the data. The differences between samples were evaluated using the Kruskal-Wallis test with multiple comparisons of average ranks, and the one-way analysis of variance (ANOVA). Tukey’s test was used as a *post hoc* test *(p* < 0.05). 

## 6. Conclusions

Our data demonstrate that TR and AtPAP1TR root extracts of *L. sibiricus* inhibited the cell viability of human CCRF-CEM and K-562 cells. Additionally, both extracts decreased mitochondrial membrane potential inducing apoptosis in K-562 and CCRF-CEM cells by causing DNA damage in *ND1, ND5* or *TP53* genes region, but most effective was the AtPAP1 TR root extract. These findings suggest that *L. sibiricus* root extracts may potentially be used as a potential therapeutic agent against cancer diseases but further in vitro and in vivo studies are needed to test this hypothesis.

## Figures and Tables

**Table 1:** Phenolic acids content in TR and AtPAP1 TR roots of *L. sibircus.* Data from our previous studies [15,17].

Phenolic Compounds	TR Extract (µg/g DW)	AtPAP1 TR Transgenic Extract from Bioreactor (µg/g DW)
**Neochlorogenic acid**	8 ± 0.4 ^a^	7 ± 1 ^a^
**Chlorogenic acid**	4104 ± 8.7 ^a^	21,520 ± 1160 ^c^
**Caffeic acid**	4176 ± 9.0 ^a^	14,500 ± 600 ^d^
**p-coumaric acid**	30 ± 0.1 ^a^	20 ± 4 ^a^
**Ferulic acid**	660 ± 27.1 ^a^	1870 ± 160 ^c^

The phenolic acids were determined in 80% aqueous methanol root extracts from TR (used as the control) and AtPAP1 TR grown in bioreactor. Different superscript letter within the rows indicates significant differences in the mean values at *p* ˂ 0.05 (the Kruskal-Wallis test).

**Table 2:** Sequence of oligonucleotides used in this study.

Genome	Target Gene	Forward Primer Sequences (5′→3′)	Reverse Primer Sequence (5′→3′)	Amplicon Length (bp)
Mitochondrial	*ND1* (mitochondrially encoded NADH: ubiquinone oxidoreductase core subunit 1)	Long fragment: ATGGCCAACCTCCTACTCCT	Long fragment: GATGAGTGTGCCTGCAAAGA	1214
		Small fragment: CCTAAAACCCGCCACATCTA	Small fragment: GCCTAGGTTGAGGTTGACCA	124
	*ND5* (mitochondrially encoded NADH:ubiquinone oxidoreductase core subunit 5)	Long fragment: TCCAACTCATGAGACCCACA	Long fragment: AGGTGATGATGGAGGTGGAG	1156
		Small fragment: AGGCGCTATCACCACTCTGT	Small fragment: TTGGTTGATGCCGATTGTAA	124
Nuclear	*TP53* (tumor protein p53)	Long fragment: GGGTGTAGATGATGGGGATG	Long fragment: AACTGCGGAATGAAACAACC	1172
		Small fragment: AAGCTGCTAAGGTCCCACAA	Small fragment: GGAAAGATCGCTCCAGGAA	56
	*HPRT1* (hypoxanthine phosphoribosyltransferase 1)	Long fragment: AGGGCAAAGGATGTGTTACG	Long fragment: AGTGGTTTCTGGTGCGACTT	1018
		Small fragment: TGCTGACCTGCTGGATTACA	Small fragment: TCTACAGTCATAGGAATGGATCTATCA	69

All primers were designed with the help of the Primer3 software and synthesized by Sigma-Aldrich.
